# Diapause Formation and Downregulation of Insulin-Like Signaling via DAF-16/FOXO Delays Axonal Degeneration and Neuronal Loss

**DOI:** 10.1371/journal.pgen.1003141

**Published:** 2012-12-27

**Authors:** Andrea Calixto, Juan S. Jara, Felipe A. Court

**Affiliations:** 1 Department of Cellular Biology, Faculty of Biology, Pontifical Catholic University of Chile, Santiago, Chile; 2 Millennium Nucleus for Regenerative Biology, Faculty of Biology, Pontifical Catholic University of Chile, Santiago, Chile; 3 NeuroUnion Biomedical Foundation, Santiago, Chile; University of California San Diego, United States of America

## Abstract

Axonal degeneration is a key event in the pathogenesis of neurodegenerative conditions. We show here that *mec-4d* triggered axonal degeneration of *Caenorhabditis elegans* neurons and mammalian axons share mechanistical similarities, as both are rescued by inhibition of calcium increase, mitochondrial dysfunction, and NMNAT overexpression. We then explore whether reactive oxygen species (ROS) participate in axonal degeneration and neuronal demise. *C. elegans* dauers have enhanced anti-ROS systems, and dauer *mec-4d* worms are completely protected from axonal degeneration and neuronal loss. Mechanistically, downregulation of the Insulin/IGF-1-like signaling (IIS) pathway protects neurons from degenerating in a DAF-16/FOXO–dependent manner and is related to superoxide dismutase and catalase-increased expression. Caloric restriction and systemic antioxidant treatment, which decrease oxidative damage, protect *C. elegans* axons from *mec-4d*-mediated degeneration and delay Wallerian degeneration in mice. In summary, we show that the IIS pathway is essential in maintaining neuronal homeostasis under pro-degenerative stimuli and identify ROS as a key intermediate of neuronal degeneration *in vivo*. Since axonal degeneration represents an early pathological event in neurodegeneration, our work identifies potential targets for therapeutic intervention in several conditions characterized by axonal loss and functional impairment.

## Author Summary

Axonal degeneration and neuronal loss are currently considered crucial pathological factors in neurodegenerative diseases. Therefore, delaying or blocking these procesess is key for neuroprotection. In this work, we used an *in vivo* approach combining invertebrate (*C. elegans*) and vertebrate (mice) model systems to identify a novel and unexpected player in the mechanisms of axonal degeneration. Here, we demonstrate that both neuronal somas and axons degenerate through a step dependent on oxidative stress that can be efficiently delayed by genetic downregulation of a pathway controlling oxidative stress resistance. Impressively, we discovered that diapause formation, which is a state related to hibernating conditions, fully prevents neuronal degeneration. We uncovered new players in the degenerative mechanisms of neurons with relevance for several conditions associated to axonal degeneration, such as multiple sclerosis, motoneuron, and Parkinson diseases, offering novel potential targets for neuroprotection.

## Introduction

Neuronal loss constitutes an irreversible end point of several neurodegenerative conditions triggered by diverse stimuli. Nevertheless, early neuronal dysfunction is associated with degeneration of neuronal processes, including axons and dendrites, causing a progressive loss of neuronal function [1], [2]. Axons are particularly susceptible to genetic and toxic insults and represent a key therapeutic target for neuroprotection [3], [4]. Degeneration of axons is non-apoptotic [5], [6], and depends on the activation of the mitochondrial permeability transition pore (mPTP and reference [7]), intra-axonal calcium rise, and calpain activation [8].

Much of our current knowledge regarding the mechanisms of axonal degeneration originates from studies in the Wld^s^ mice, which show delayed axonal degeneration caused by mechanical injuries, hypoxia and other toxic stimuli [5]. The Wld^s^-dependent protection of axons is given by overexpression of a chimera between nuclear NMNAT1 and a non-catalytic sequence from a ubiquitin ligase [9]. Importantly, slowing down axonal degeneration by Wld^s^ expression in mouse models of neurodegenerative conditions delays the disease progression and severity [10]–[12], pointing out to a crucial role of axonal degeneration in diseases of varied nature.

Despite the advancement in the identification of molecular players and pathways implicated in axonal degeneration, how they integrate into a degenerative process triggered by a wide variety of conditions remains inconclusive and needs further examination *in vivo*. Therefore it is important to characterize axonal degeneration in tractable model systems such as *C. elegans*, which are suitable for genetic and pharmacological interventions with functional and morphological readouts of neuronal integrity.

In *C. elegans*, constitutively active degenerins cause degeneration of neuronal somas and axons by ultrastructural and macroscopical features reminiscent of excitotoxic neuronal death in mammals [13]. Non-apoptotic death of degenerin-expressing *C. elegans* neurons involves intracellular calcium rise, activation of intracellular proteases and autophagy [14]–[16], and is currently considered to occur by necrosis, also responsible for cell death in stroke, brain trauma and other human neuropathological conditions [17]. Key players in the execution of necrosis are mitochondrial dysfunction and oxidative stress [17], but the role that intracellular ROS generation plays in axonal degeneration has not been addressed *in vivo*.

The cell is equipped with a homeostatic network to cope with oxidative stress in physiological and pathological situations. One of the master regulators of this homeostatic response to oxidative stress is the Insulin/IGF-1-like signaling (IIS) pathway, which has been associated with the protection of diverse tissues from chemical and genetic insults [18].

Here, we show for the first time that axonal and neuronal degeneration triggered by the hyperactivated degenerin channel MEC-4d, can be prevented by diapause entry and by increasing the anti-oxidative capacity of the neuron. Mechanistically, this increase is mediated by the IIS pathway in a DAF-16/FOXO dependent manner and relies on the activity of the antioxidant enzymes superoxide dismutases (SODs) and catalases. In addition to including the axonal compartment in our analyses, we performed a functional assessment of neuronal integrity to evaluate if prevention of neurodegeneration rendered functional neurons [19], generating a powerful model for mechanistic enquiries. Neuronal function is a critical aspect to be evaluated in therapeutic applications and a readout missing from commonly used models. Importantly, we demonstrate that *mec-4d*-dependent degeneration can be prevented morphologically and functionally by mPTP inhibition and NMNAT overexpression, which delay axonal degeneration in mammals [7], [9]. Finally, we extend these findings to mice and demonstrate that axonal degeneration can be strongly delayed *in vivo* by systemic manipulation of the cellular antioxidant capacity. In summary, our work shows that prevention of neuronal degeneration and functional loss can be achieved by a systemic shift of metabolic state leading to an increase in cellular anti-oxidative defense systems.

## Results

### Morphological and functional time course of MEC-4d mediated degeneration of somas and axons

**Figure 1:**
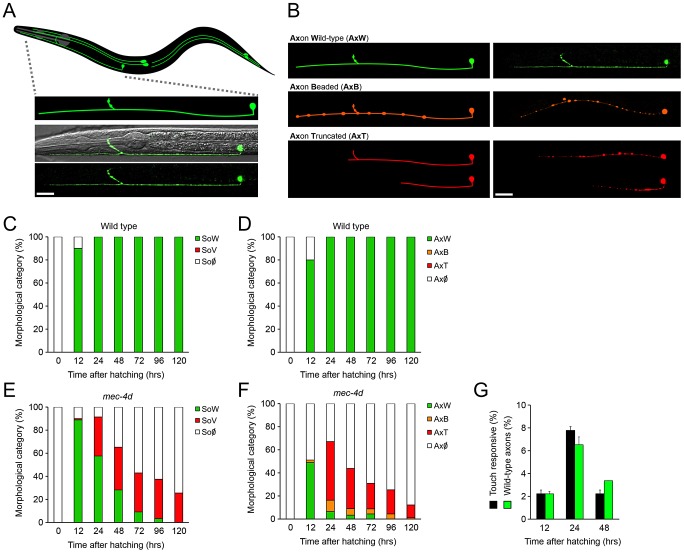
Functional and morphological time course of *mec-4d* mediated degeneration of *C. elegans* AVM neuron. (A) Schematic representation of the six mechanosensory neurons of an adult *C. elegans* with an expanded representation of the AVM neuron (anterior part of the worm is to the left) and below a confocal image of the AVM expressing *gfp* under a touch neuron specific promoter (*P_mec-17_mec-17::gfp*) in a wild type (N2) background merged with a Nomarski optic micrograph. Only the anterior portion is shown. Scale bar, 25 µm. (B) Morphological categories of the AVM axon in a wild type condition and during the progression of degeneration triggered by the expression of the MEC-4d degenerin channel. Schematic representations of the different axonal morphological categories are shown in the left panel, the right panel shows neurons as seen by confocal microscopy. GFP-expressing neurons have been pseudo-colored to match the morphological categories. Scale bar, 25 µm. (C–F) Temporal analysis of morphological categories from the time of hatching for AVM somas and axons in wild-type (C–D), and *mec-4d* mutants (E–F). (C) AVM somas in the wild type strain (*P_mec-17_mec-17::gfp*) are almost fully present at 12 hours. (D) AVM axons are still growing at 12 hours but at 24 hours they have reached their full extension. (E) AVM somas in *mec-4d* mutants appear by 12 hours and therein steadily degenerate, going through a vacuolated stage. (F) AVM axons in *mec-4d* mutants appear and extend along the anterior region at 12 hours and thereafter they degenerate through beaded and then truncated intermediate stages (mean value for each category is shown; error bars [<0.1%] are not included; N = 3 of 30 worms each per group). (G) Anterior touch response (black bars) and percentage of morphologically wild-type axons (green bars) of *mec-4d* worms at 12, 24 and 48 hours post hatching. For the 12-hour time point of this graph, only AVM neurons with fully-grown axons were considered as wild-type (mean values are shown; error bars indicates SEM; N = 3 of 30 worms each per group). SoW: soma wild type; SoV: Soma vacuolated; So∅: soma absent; AxW: Axon wild type; AxB: Axon beaded; AxT: Axon truncated; Ax∅: Axon absent.

The use of *C. elegans* to systematically study axonal degeneration has not been explored so far. *C. elegans* touch receptor neurons (TRNs) expressing the constitutively open MEC-4d degenerin channel, constitute an ideal model for axonal degeneration as the pro-degenerating stimuli is endogenously triggered (*mec-4d* expression) and its degeneration and protection can be assessed morphologically and by loss of touch sensitivity. From the six TRNs (ALML, ALMR, PLML, PLMR, AVM and PVM; see Figure 1A), we investigated the AVM neuron because it arises post-embryonically, thus the degenerative process can be observed from the beginning. Additionally, the AVM by itself gives a functional response to anterior touch [19], as when AVMs arise, the other neurons of the anterior touch circuit, the ALMs, have already degenerated (data not shown). To assess the AVM neuron morphologically, we used a strain that expresses *gfp* under a TRN specific promoter (*P_mec-17_mec-17::gfp*) in wild type or *mec-4d(e1611)* worms. AVM neurons in *mec-4d* mutants, appeared and extended axons that reached similar sizes to wild type AVM neurons. Thereafter, AVM somas and axons followed a stereotyped form of degeneration (Figure 1B). First, full length axons become beaded and later become truncated from the most distal end (Figure 1B). Somas become vacuolated and later disappear (Figure 1B; see Methods for a description of categories). This order of events for AVM soma and axon degeneration in *mec-4d* mutants is consistent with previous qualitative electron microscope analysis [13].

We performed a thorough temporal analysis of the birth and degeneration of AVM somas and axons by using *gfp* expression as a reporter. In wild type worms, most AVM somas and axons appear 12 hours post hatching; 24 hours after hatching, all axons have reached their full length (Figure 1C and 1D). In *mec-4d* mutants, 89% of somas have appeared at 12 hours, and around 48.9% axons are intact (Figure 1E and 1F). Thereafter, AVM axons degenerate as described above (Figure 1B). By 24 hours after hatching, only 7% of axons remain intact (Figure 1F), and 58% of somas appear healthy (Figure 1E). 72 hours post hatching, both somas and axons have nearly completely degenerated (Figure 1E and 1F).

We performed touch tests to *mec-4d* mutants at different times after hatching. The percentage of anterior-touch responsive worms at each time point correlates well with AVMs in the axon wild type (AxW) category (Figure 1G). At 12 hours after hatching, *mec-4d* animals do not respond because the AVB interneuron, which connects the AVM to motoneurons has not yet arisen [20].

To evaluate our genetic and pharmacological interventions on *mec-4d* mutants we scored at 72 hours after hatching, when AVM neurons have almost completely degenerated (Figure 1E and 1F), hence an adequate time point to observe unequivocal neuronal protection. Additional relevance comes from the fact that at 72 hours after hatching, worms have reached adulthood and have completed their life cycle.

In summary, we have introduced two novel elements in the model of degeneration triggered by *mec-4d*: we included the axonal compartment and performed functional analyses of neuronal integrity, which allows a behavioral evaluation of treatments that delay or prevent neuronal degeneration.

### Diapause entry prevents neuronal degeneration

Upon harsh conditions, *C. elegans* larvae enter a diapause state called dauer, from where they resume development once the environment becomes favorable [21]. Among other stress resistant changes, dauers have enhanced anti-oxidative defense systems [22]–[25] due to downregulation of the IIS pathway, which leads to the activation of transcription factors DAF-16/FOXO [26] and SKN-1/Nrf-2 [27]. DAF-16 targets genes like *sod-3* and *ctl-1* [28], while SKN-1/Nrf-2 mobilizes the phase 2 detoxification response [29], [30].

**Figure 2:**
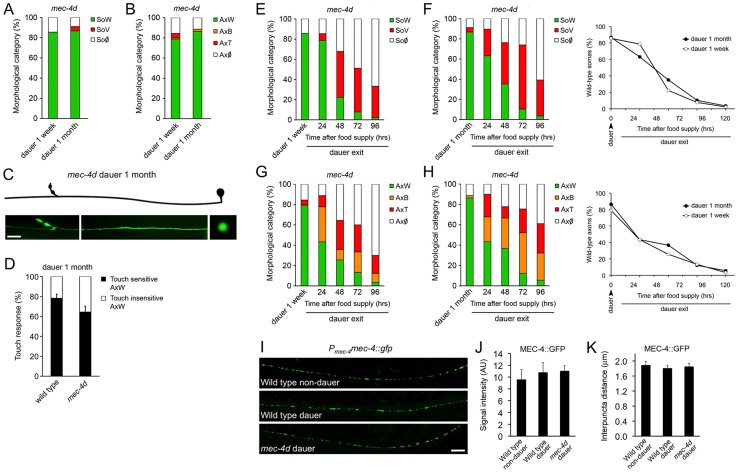
*C. elegans* dauer state prevents neuronal degeneration triggered by *mec-4d*. *mec-4d* dauer worms were scored by morphological and functional readouts. Both AVM somas (A) and axons (B) were protected from degeneration when worms were kept in a dauer state for one week or one month (mean value for each category is shown; error bars [<0.1%] are not included; N = 3 of 30 worms each per group). (C) Morphologically conserved AVM neuron from a *mec-4d* mutant kept in diapause for 1 month. Scale bar, 10 µm. (D) Anterior touch response of *mec-4d* mutants kept in diapause for one month. Both wild-type (N2) and *mec-4d* groups have wild-type somas and axons as studied after the touch test by fluorescent microscopy. An equal fraction in both groups is unresponsive, which represents a dauer-dependent trait (mean values are shown, error bars indicates SEM, N = 3 of 20 worms each per group). (E–H) Degeneration of somas and axons during dauer exit by food reposition takes place in a rate that is independent to the dauer length. Line graphs in F and H (right side), represent the rate of decay of wild type somas (F) and axons (H) during dauer exit (mean value for each category is shown; error bars [<0.1%] are not included; N = 3 of 30 worms each per group; Student's t test at each time point was performed for SoW and AxW categories, no significant differences were found between groups). (I) In *uIs58* wild-type non-dauers and dauers expressing MEC-4::GFP *(P_mec-4_mec-4::gfp)*, the GFP signal was expressed in a punctate pattern along axons, as in animals with the *mec-4d* (*e1611*) mutation. Scale bar, 10 µm. Quantification of MEC-4::GFP signal intensity (J) and interpuncta distance (K) reveal no significant differences between conditions (N = 9 worms per group; analyzed by Student's t test compared to wild-type non-dauer values).

To examine whether neuronal degeneration is hampered by diapause entry, *mec-4d* worms were induced to form dauer by starvation and kept in diapause for a week or a month. Strikingly, there was an almost complete prevention of somatic and axonal degeneration by morphological criteria in *mec-4d* dauer, independently of the diapause length (Figure 2A–2C). Furthermore, AVM neurons of one-month old *mec-4d* dauers responded to the anterior touch (Figure 2D). In one-month old wild type dauers, 21.9% were anterior and posteriorly touch insensitive (Figure 2D). Considering this percent of insensitive wild type dauers, there is a precise correlation between morphologically wild type axons of *mec-4d* AVMs and their ability to respond to touch. Noticeably, this protection is stronger than most other genetic or pharmacological treatment reported previously for the *mec-4d* stressor, and additionally includes a functional readout. Dauer *mec-4(u253)* loss of function mutants were largely insensitive to touch (2 out of 90 responded to two of ten touches), demonstrating that touch response during dauer is MEC-4 dependent.

We then examined if the protection conferred by dauer was reversed upon exit from this state. For this purpose, dauer worms were placed on food and AVM integrity was scored every 24 hours for 120 hours. Degeneration of somas and axons during dauer exit resumes in a dauer length-independent fashion (Figure 2E–2H).

To rule out that AVM protection during dauer was due to a reduction of *mec-4d* expression in diapause, we followed MEC-4::GFP expression by means of the *uIs58* transgene (*P_mec-4_mec-4::gfp*) in dauer and non-dauer wild type animals. The MEC-4 channel is patterned in regularly spaced puncta along the TRN axons (Figure 2I and reference [31]), allowing the quantification of both intensity by *gfp* expression and the interpuncta distance, which reflects the distribution of the mechanosensory channel. The same criteria was used to assess whether in *mec-4d* dauers the expression of MEC-4 was impaired or diminished by the *mec-4d* (*e1611*) dominant mutation. Dauer entry did not affect MEC-4 expression nor did the *mec-4d* (*e1611*) mutation, as GFP intensity was similar in all three conditions (Figure 2I and 2J). Importantly, the interpuncta distance was also unchanged in both wild type and *mec-4d* animals compared to non-dauer wild type worms (Figure 2I and 2K), confirming that the distribution of the mechanosensory channel is not affected neither by the *mec-4d* mutation nor by dauer entry. In addition, both wild type and *mec-4d* dauers respond to anterior gentle touch (Figure 2D), strongly suggesting that MEC-4 is expressed and functional.

**Figure 3:**
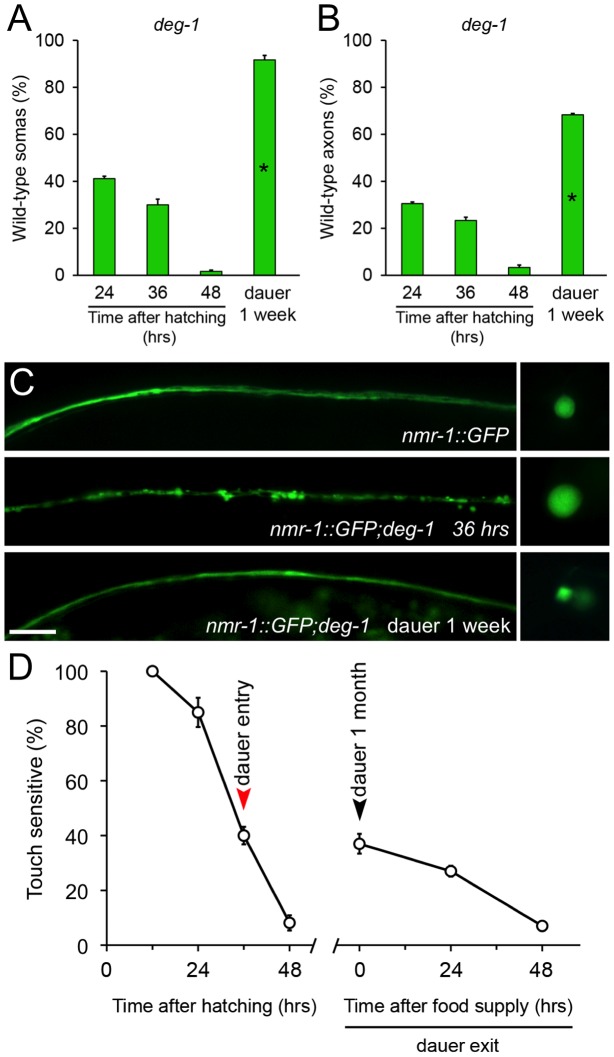
Diapause entry prevents degeneration of the PVC neurons triggered by the *deg-1(u38)* mutation. *deg-1(u38)* dauer worms were scored by morphological and functional readouts. Both PVC somas (A) and axons (B) were protected from degeneration when worms were kept in diapause for one week (mean value is shown; error bars indicate SEM; N = 3 of 10 worms each per group; *p<0.01 by Student's t test compared with 24 hours after hatching). (C) Morphology of the PVC neuron observed by expression of *nmr-1::gfp* on wild type (top), *deg-1(u38)* mutants at 36 hours (middle) and *deg-1(u38)* dauers (bottom). Scale bar, 10 µm. (D) Posterior touch responses of *deg-1(u38)* mutants after hatching, kept as dauers for one month and during dauer exit by food reposition. Only worms that responded to the anterior touch were included (mean values are shown, error bars indicate SEM, N = 3 of 33 worms each per group).

To study whether this phenomenon was extensive to other cells and pro-degenerative stimuli, we tested whether dauer entry prevented degeneration of PVC neurons caused by the *deg-1(u38)* mutation. Degeneration of the PVC renders worms insensitive to posterior touch [32]. To observe degeneration of somas and axons caused by the *deg-1(u38)* dominant mutation, we created a strain that expresses *gfp* under the *nmr-1* promoter (*P_nmr-1_nmr-1::gfp*, reference [33]) in a *deg-1 (u38)* mutant background. At the time corresponding to dauer entry, 30% of PVC somas and 23% of axons were wild type (Figure 3A and 3B). We induced *deg-1(u38)* animals to form dauer for one month and analyzed the morphology of their PVC neurons and performed a posterior touch test. Impressively, 91% of somas and 68% of axons remained wild type in dauer, and the protection of touch sensitivity was also maintained (Figure 3A–3D), and decayed as worms exited this stage by food reposition (Figure 3D). These results demonstrates that protection conferred by dauer to degenerating neurons is independent of cell type and stimuli, and persists as long as animals remain in diapause.

### IIS operates cell autonomously to delay neuronal degeneration

**Figure 4:**
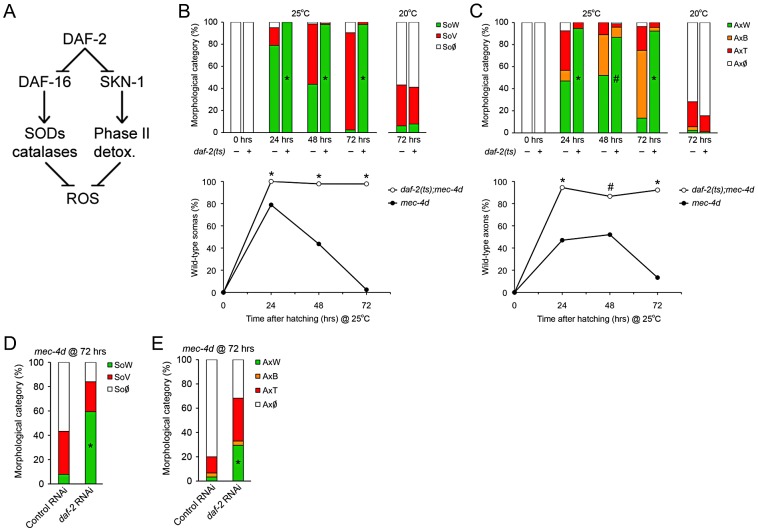
Downregulation of Insulin/IGF-1-like signaling (IIS) prevents neuronal degeneration triggered by *mec-4d*. (A) DAF-2 negatively regulates both DAF-16/FOXO and SKN-1/Nrf2, which by different mechanisms increases the cellular antioxidative capacity. (B–C) The possible protective role of the IIS pathway activation in *mec-4d* degeneration was explored in *daf-2(e1370ts)* mutants. At the restrictive temperature both somas (B) and axons (C) were significantly protected respect to the controls not carrying the *mec-4d(e1611)* mutation or *daf-2(e1370ts)* mutants carrying *mec-4d(e1611)* and raised at 20°C. Below each bar graph, a line graph incorporating only the SoW and AxW of *daf-2;mec-4d* vs. *mec-4d* raised at 25 °C is shown. Notice that *mec-4d*-dependent degeneration of both somas and axons is delayed for about one day at 25°C compared to 20°C (mean value for each category is shown; error bars [<0.1%] are not included; N = 3 of 30 worms each per group; ^#^p<0.05 and *p<0.01 by Student's t test compared with control at the same temperature for the SoW and AxW category). (D–E) TRN-autonomous *daf-2(RNAi)* was performed in the *P_mec-17_mec-17::gfp* strain carrying the *mec-4d(e1611)* mutation (WCH6). Significant morphological protection of both AVM somas (D) and axons (E) is seen at 72 hours post hatching (mean value for each category is shown; error bars [<0.1%] are not included; N = 3 of 30 worms each per group; *p<0.001 by Student's t test compared with control at the same temperature for the SoW and AxW category).

The *C. elegans* insulin receptor DAF-2 negatively controls DAF-16/FOXO and SKN-1/Nrf-2 activity, and *daf-2* deficient worms share with dauers similar transcription signatures leading to an increase in expression of antioxidant enzymes (Figure 4A and references [22], [27]). We wanted to dissect out the DAF-2 dependent components of dauer that protects *mec-4d* neurons from degeneration. To this end, we generated a strain with a temperature sensitive mutation in *daf-2(e1370ts)*, together with *mec-4d(e1611)* and a fluorescent marker in TRNs (*P_mec-17_mec-17::gfp*). At 25°C, DAF-2 is not functional in *daf-2(e1370ts)* mutants and nuclear translocation of DAF-16 and SKN-1 activates their target genes [30], [34]. At 25°C, *mec-4d* AVMs appear and degenerate and by 72 hours almost no axons or somas remain (Figure 4B and 4C). In contrast, raising *daf-2(e1370ts);mec-4d(e1611)* at 25°C from the time of hatching confers an almost complete protection to both axons and somas similar to the provided in dauer (Figure 4B and 4C).

Insulin pathway in *C. elegans* mediates life span extension, dauer formation and stress resistance by systemic and cell autonomous mechanisms [35]–[38]. We wanted to know if cell autonomous downregulation of DAF-2 in TRN prevents *mec-4d*-dependent TRN degeneration. To this end, we created a systemic RNAi defective strain with TRN-enhanced RNAi also carrying the *mec-4d(e1611)* mutation and a fluorescent marker in touch neurons (*P_mec-17_mec-17::gfp*, WCH6; see Methods). We fed *daf-2* and control *unc-22(RNAi)* to newly hatched nematodes and scored AVMs 72 hours later. As expected, *unc-22(RNAi)* treated animals shown no twitching phenotype, since systemic RNAi is impaired in this strain. Impressively, TRN-autonomous *daf-2(RNAi)* prevented AVM degeneration, rendering about 69% somas and 33% axons in a wild type category, compared to 8% and 3% in control RNAi (Figure 4D and 4E). Taken together our data suggest that repression of the IIS pathway leads to prevention of neuronal demise triggered by *mec-4d* and that downregulation of the IIS only in TRN is sufficient to provide this protection.

### DAF-16 contributes to inhibition of neuronal degeneration associated with reduced IIS signaling

**Figure 5:**
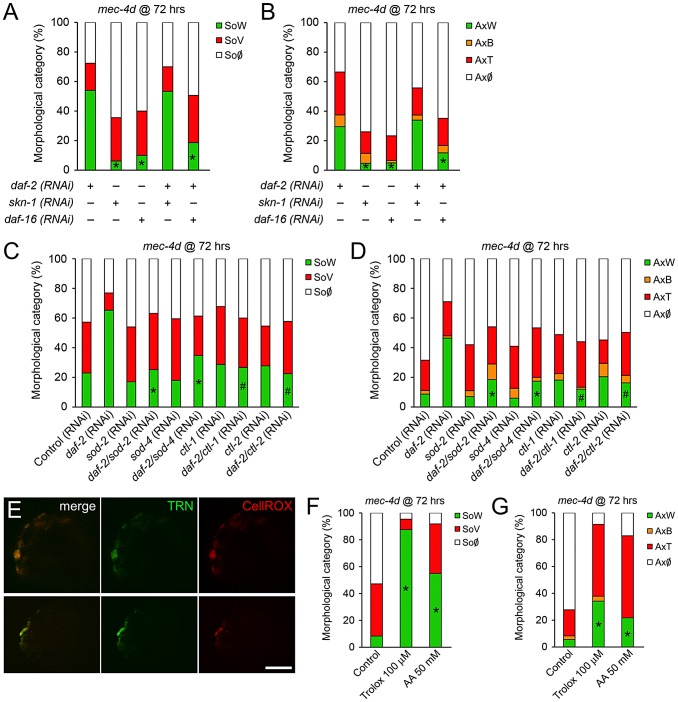
DAF-16 contributes to inhibition of *mec-4d*–dependent AVM degeneration associated with reduced IIS signaling. (A–B) Double RNAi experiments in the TRN-autonomous RNAi strain carrying the *mec-4d(e1611)* mutation (WCH6) were performed to assess the role of DAF-16 and SKN-1 downstream DAF-2 in the protection of AVM neurons. In both somas (A) and axons (B), single *daf-2(RNAi)*, but not *skn-1* or *daf-16*, protects the AVM neuron from degeneration. When combined, only *daf-16(RNAi)* is able to revert the protective phenotype given by *daf-2(RNAi)* (mean value for each category is shown; error bars [<0.1%] are not included; N = 3 of 30 worms each per group; *p<0.05 by Student's t test compared to single *daf-2(RNAi)* for the SoW and AxW category). (C–D) Combinatorial RNAi experiments were performed to address the role of endogenous antioxidant enzymes. *daf-2(RNAi)* together with either *sod-2*, *sod-4*, *ctl-1* or *ctl-2* were fed to the TRN-autonomous RNAi strain carrying the *mec-4d(e1611)* mutation (WCH6). All double RNAi significantly decreased the *daf-2(RNAi)* protection of degeneration of somas and axons of the AVM neuron. (E) ROS was visualized using the fluorogenic dye CellROX (red) in *mec-4d* embryos. A strong signal of the dye co-localized with the endogeneous GFP expressed by degenerating TNRs. Scale bar, 5 µm. (F–G) *mec-4d* worms were treated from the time of hatching with the ROS scavenger trolox (100 µm) and ascorbic acid (AA, 50 mM); after 72 hours both somas (F) and axons (G) were protected several fold compared to control nematodes (mean value for each category is shown; error bars [<1%] not included; N = 3 of 30 worms each per group; *p<0.005 by Student's t test compared with control for the SoW and AxW category).

To define the contribution of DAF-16 and SKN-1 in the prevention of *mec-4d*-dependent neuronal degeneration by DAF-2 downregulation, we performed double RNAi of *daf-2* together with *daf-16* or *skn-1* TRN-autonomously. Combinatorial RNAi has been shown extensively to be an efficient tool to target two genes concomitantly [39]. *daf-2/skn-1(RNAi)* resembled the protection that *daf-2(RNAi)* alone conferred to axons and somas while *daf-2/daf-16(RNAi)* reverted this protection (Figure 5A and 5B). This result suggests that elevation in DAF-16, but not SKN-1 targets in TRN is responsible for neuronal protection after DAF-2 downregulation. No effects in degeneration of somas or axons triggered by *mec-4d* were observed when *skn-1* or *daf-16(RNAi)* were performed alone (Figure 5A and 5B).

To rule out that the effect observed in *daf-2/skn-1(RNAi)* was due to inefficient *skn-1(RNAi)*, we fed bacteria expressing *skn-1* dsRNA to *mec-4d* worms to reveal the embryonic lethal phenotype that has been reported for this gene (www.wormbase.org). More than 90% of *mec-4d* embryos on *skn-1(RNAi)* did not hatch, confirming the expected phenotype. Additionally, it is unlikely that the lack of effect of *daf-2/skn-1(RNAi)* on neuronal protection is due to an artifact of double RNAi inefficiency since *daf-16* combined with *daf-2(RNAi)* successfully reverted the *daf-2(RNAi)* protection.

Key enzymes that control oxidative stress downstream of DAF-2 are superoxide dismutases and catalases [40], which are compartmentalized within the cell to distinct organelles. To directly test whether antioxidant endogenous defenses are needed for the protection conferred by downregulation of the IIS pathway, we performed combinatorial TRN-autonomous RNAi of *daf-2* with *sod-2*, *sod-4*, *ctl-1* or *ctl-2*. Protection of AVM somas and axons by *daf-2(RNAi)* was reverted by *sod-2*, *sod-4*, *ctl-1* and *ctl-2(RNAi)* in somas and axons (Figure 5C and 5D). These results strongly argue that protection of neuronal degeneration by downregulation of DAF-2 relies on the activity of antioxidant enzymes, supporting a role for ROS in neuronal degeneration triggered by MEC-4d.

### Antioxidants prevent neuronal degeneration

Heightened antioxidant capacity of worms by activation of DAF-16/FOXO after *daf-2* downregulation, dramatically prevented somatic and axonal degeneration of AVM touch neurons in *mec-4d* mutants. Importantly, TRNs in *mec-4d* animals were positive for a fluorogenic ROS-dependent dye (Figure 5E), demonstrating a heightened oxidative stress state in TRNs during the degenerative process. We then tested whether the ROS scavengers trolox, a vitamin E derivative, and ascorbic acid (AA) acutely protect the AVM from *mec-4d* dependent degeneration. Trolox and AA treatment from the time of hatching greatly protected AVM somas and axons from degeneration at 72 hours (Figure 5F and 5G). Protection of neuronal somas was almost complete, from 8% of worms with wild type somas in controls (no treatment) to 88% and 55% in trolox and AA-treated worms, respectively (Figure 5F). Axons were also protected, with 6% of worms with intact axons in controls, rising to 34% and 22% under trolox and AA treatment, respectively (Figure 5G).

From these results, we conclude that elevated ROS is a key intermediate pathway for neuronal degeneration triggered by MEC-4d stimuli, and that degeneration of both somas and axons can be prevented by systemic antioxidant treatment.

### Dietary restriction prevents neuronal degeneration in *C. elegans*

**Figure 6:**
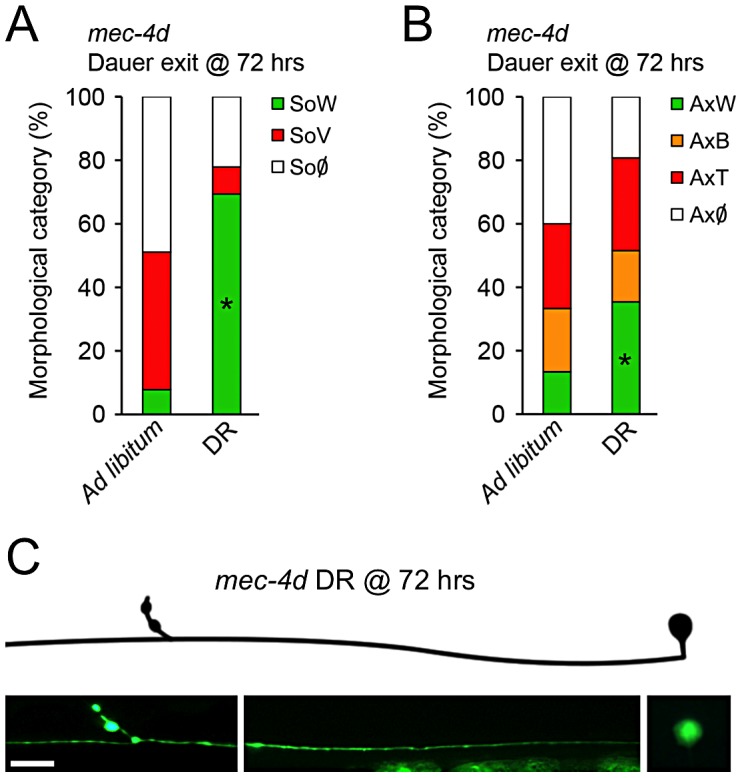
Dietary restriction inhibits *mec-4d*–dependent neuronal degeneration in *C. elegans*. (A–B) The influence of dietary restriction (DR) in TRN degeneration was investigated by fasting *mec-4d* worms intermittently with a protocol consisting of 3 hours of *ad libitum* food regime followed by 9 hours of starvation. After 72 hours, the worms on DR show a significant protection of both AVM somas (A) and axons (B) compared to the 72 hours *ad libitum* fed worms (mean value for each category is shown; error bars [<1%] not included; N = 3 of 30 worms each per group; *p<0.005 by Student's t test compared with control for the SoW and AxW category). (C) AVM neuron with a morphologically conserved soma and axon from a *mec-4d* worm subjected to DR for 72 hours by means of intermittent fasting. Scale bar, 10 µm.

Animals fed with a caloric restricted diet have reduced oxidative stress levels, and this is proposed as one of the underlying causes for life span extension and delay in age-associated conditions [41]. Hence, we wondered if dietary restriction also impacted degeneration of the AVM in *mec-4d* worms. We designed an intermittent fasting protocol during dauer exit as *mec-4d* dauers have protected AVMs and are developmentally synchronized. To this end, *mec-4d* dauers (Figure 2A–2D) were exited from diapause by a 3 hour food period followed by 9 hours without food (see Methods). This schedule was repeated until 72 hours and AVM integrity was compared to dauers exiting in an *ad-libitum* food regime. Importantly, nematodes under intermittent fasting developed normally and were fertile when they reached adulthood (data not shown). Dietary restriction conferred a strong protection of 69% to somas and 35% to axons, compared to 7% and 13% in the *ad libitum* regime, respectively (Figure 6A–6C). This demonstrates, that dietary restriction, a treatment that lowers oxidative stress, protects the TRN from degenerating.

### MEC-4d and mammalian axonal degeneration are mechanistically similar

**Figure 7:**
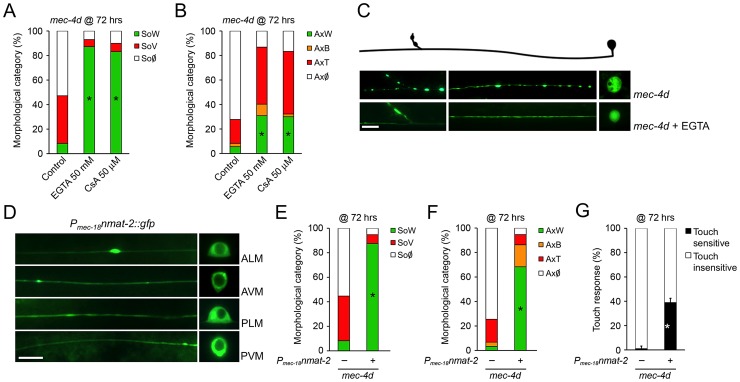
*C. elegans* and mammalian neurons share degenerative mechanisms triggered by diverse pro-degenerative stimuli. The mechanisms associated to somatic and axonal degeneration of AVM-*mec-4d* neurons were studied by pharmacological and genetic means. Worms were grown for 72 hours after hatching in food supplemented with EGTA (50 mM) or cyclosporin A (CsA, 50 µM). Both drugs delay degeneration of AVM somas (A) and axons (B) several fold (mean value for each category is shown; error bars [<1%] not included; N = 3 of 30 worms each per group; *p<0.01 by Student's t test compared with control for the SoW and AxW category). (C) Fluorescent micrograph of a beaded axon (AxB) and a vacuolated soma (SoV) of a *mec-4d*-expressing AVM, contrasted with an AVM neuron treated with EGTA displaying a wild-type axon (AxW) and soma (SoW). Scale bar, 10 µm. (D) Touch neuron-specific expression of *nmat-2::gfp* (*P_mec-18_nmat-2::gfp*), which is restricted to the cytosol in all TRNs. Scale bar, 10 µm. Overexpression of NMAT-2::GFP in TRN protects to a large extent somas (E) and axons (F) from *mec-4d* dependent degeneration at 72 hours post hatching (mean value for each category is shown; error bars [<0.1%] are not included; N = 3 of 30 worms each per group; *p<0.01 by Student's t test compared with control for the SoW and AxW category). (G) Anterior touch response of worms at 72 hours post hatching. The almost complete functional impairment triggered by *mec-4d* is significantly rescued by NMAT-2::GFP overexpression (mean values are shown, error bars indicates SEM, N = 3 of 30 worms each per group; p<0.05 by Student's t test for touch sensitivity values).

Ideally, the knowledge we gather from a simple model organism as *C. elegans* should be translated into a mammalian animal model. To this end, we tested if *mec-4d* triggered neuronal degeneration proceeds by similar mechanisms to degeneration of mammalian neurons. As reported only for somas [14], supplementing EGTA to *mec-4d* mutants from the time of hatching also significantly reduces degeneration of AVM axons at 72 hours (Figure 7A–7C), as in mammals [8]. Second, it has been shown that axonal degeneration in mammalian neurons requires mPTP activation [7], which is pharmacologically inhibited by cyclosporin A (CsA). CsA treatment strongly reduces both soma and axon demise (Figure 7A and 7B). Finally, delay of axonal degeneration by NMNAT overexpression after diverse stimuli constitutes a molecular signature of non-apoptotic neuronal degeneration in several animal models, including mice, rats, flies and zebrafish [9], [42]–[46]. We asked if overexpression of *C. elegans* NMNAT protected against *mec-4d*-triggered neurodegeneration. To this end, we expressed F26H9.4, one of the two *C. elegans* NMNAT genes, fused to *gfp* under the TRN specific *mec-18* promoter. As expression of the F26H9.4 protein is restricted to the cytosol (Figure 7D), like NMNAT-2 in mammalian cells, and is similar in sequence (data not shown), we called it NMAT-2. Overexpression of NMAT-2::GFP in TRN strongly protected AVM somas and axons against *mec-4d* mediated degeneration at 72 hours post-hatching (Figure 7E and 7F). Importantly, AVM function was also rescued by NMAT-2 overexpression when the touch response was evaluated (Figure 7G). These results demonstrate that *C. elegans* neurons degenerate by similar mechanisms to mammalian neurons giving a strong base for extending our findings to a mouse model of neuronal degeneration.

### Systemic antioxidant treatment and intermittent fasting delay axonal degeneration in mice

**Figure 8:**
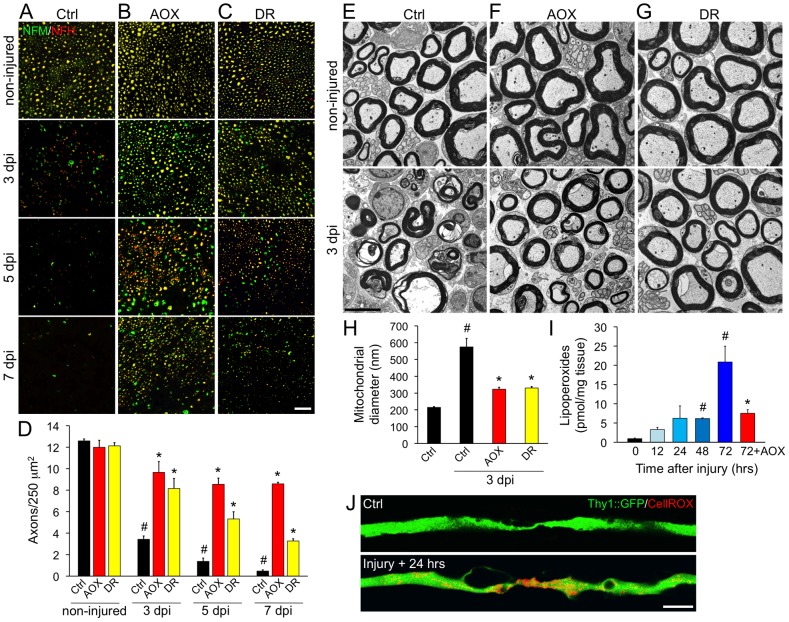
Systemic antioxidant treatment and intermittent fasting delay axonal degeneration in mice. Wild-type mice (C57BL/6J strain) were subjected to systemic treatments of the antioxidant ascorbic acid (AOX) or to dietary restriction (DR) for 10 days. After this, a sciatic nerve injury was performed unilaterally at the sciatic notch level. The degree of axonal degeneration was quantitatively assessed by immunofluorescence using neurofilament antibodies (A–D) and also studied by ultrastructural examination (E–J). (A–C) Transverse sections of sciatic nerves stained for NFM (green) and NFH (red) isoforms in control (upper panels) and 3, 5 and 7 days after injury, distal to the injury region (lower panels). A nerve crush was performed for 3 days experiments and a nerve cut for experiments lasting for 5 and 7 days, to avoid the interference of axonal regeneration in the analysis. The neurofilament signal decrease progressively at 3, 5 and 7 days after nerve injury in non-treated mice (A). Both AOX or DR treatments (B and C, respectively) strongly delays axonal degeneration triggered by nerve injury when observed 3 and 5 days after damage and AOX treatment strongly delays degeneration even at 7 days post injury. Scale bar, 20 µm. (D) Quantification of NFH-positive axons in nerve cross sections as shown in (A–C), expressed as axonal density. In injured nerves, statistically significant protection from axonal degeneration was seen after AOX and DR treatment compared to non-treated mice in all days post-damage (mean values are shown, error bars indicate SEM, N = 4 per group; #p<0.001 by Student's t test compared with non-injured controls; *p<0.005 by Student's t test compared with the respective day post-injury in non-treated mice). Representative electron micrographs (EM) of sciatic nerves from non-treated (E), AOX-treated (F) and DR-treated (G) wild-type mice. Micrographs of non-injured nerves are shown in the upper panels and from nerves 3 days after sciatic nerve crush are shown in the lower panels. In non-injured nerves from all conditions, axons are rounded, myelin sheaths are well preserved and the tissue is well organized. After nerve crush, most axons are degenerated and the myelin sheaths appear collapsed (E, bottom panel). In contrast, crushed nerves from mice systemically treated with AOX or with a restricted dietary intake by intermittent fasting, display preserved axons with conserved myelin sheaths (F and G, lower panels). Scale bar, 5 µm. (H) Diameters of axonal mitochondria were measured in EM transverse sections. The almost three-fold increase in mitochondrial diameter in crushed and non-treated nerves was prevented by both AOX and DR treatment (mean values are shown, error bars indicate SEM, N = 75–100 mitochondria/nerve of 3 mice per condition; #p<0.0001 by Student's t test compared with non-injured controls; *p<0.0001 by Student's t test compared with 3 days after crush in non-treated mice). (I) ROS-dependent lipoperoxidation measured in untreated sciatic nerve explants at 0, 12, 24, 48 and 72 hours post-injury and in nerves treated with the ROS scavenger trolox (1 mM) for 72 hours. Lipoperoxide levels progressively increase after nerve damage and AOX treatment strongly inhibit injury induced lipid peroxidation (mean values are shown, error bars indicate SEM, N = 4 per group; #p<0.05 and *p<0.01 by Student's t test compared with time 0; the AOX values are compared statistically with 72 hours non-treated nerves). (J) *In situ* ROS detection in YFP-expressing axons (green) using the vital dye CellROX (red). Uninjured axons have ROS levels not detectable by this technique, which increases considerable at 24 hours post damage. Scale bar, 5 µm.

We investigated if systemic antioxidant treatment (AOX) has an effect in Wallerian degeneration in mice, a well established model of axonal degeneration triggered by axotomy, and delayed by NMNAT overexpression [5]. Wild-type mice (C57BL/6J strain) were supplemented with ascorbic acid in the drinking water for 10 days before a nerve injury was performed in the sciatic nerve. After 3, 5 and 7 days, the degree of axonal degeneration distal to the injury site was quantified. In non-treated mice, axons disconnected from their somas degenerate to a large extent in 3 days as seen by the almost complete disappearance of neurofilament markers (Figure 8A and 8D). AOX treatment strongly protected axons from degeneration over 3, 5 and even 7 days (Figure 8B and 8D). Ultrastructurally, control sciatic nerves display mixed populations of conserved myelinated and non-myelinated axons. Three days after damage, distal nerves exhibit degenerating axons and collapsed myelin sheaths (Figure 8E). Consistent with the immunofluorescence results, AOX treatment robustly protects axons from axotomy-dependent degeneration when examined at the EM level (Figure 8F).

We then assessed if dietary restriction replicate in mice the protective effects obtained in *mec-4d* worms. To this end, mice were subjected to intermittent fasting by means of *ad libitum* feeding every other day for 10 days. Subsequently, sciatic nerves were injured and analyzed 3, 5 and 7 days later as described above. Impressively, dietary restriction in mice delays axonal degeneration at 3, 5 and 7 days after axotomy as seen both by immunofluorescence (Figure 8C and 8D) and ultrastructural analyses (Figure 8G). Ultrastructurally, the reported increase in axonal mitochondria diameter at 3 days post-axotomy (Figure 8H and reference [7]) was prevented by both AOX and dietary restriction (Figure 8H). In contralateral, non-damaged nerves, neither AOX nor DR treatments affect axonal parameters as density or ultrastructural characteristics (Figure 8B–8D, 8F and 8G).

Due to the robust protection over injury-induced axonal degeneration conferred by AOX, we wanted to establish the dynamics of oxidative stress after nerve damage. To this end, we measured lipid peroxidation, a widely used reporter of oxidative modifications [47]. Injured sciatic nerves kept *ex vivo* showed a gradual increase in lipid peroxidation over time (Figure 8I), and at 3 days post injury, lipoperoxide levels increased about 20-fold compared to non-injured sciatic nerves (Figure 8I). Importantly, this increase in lipid peroxidation was significantly prevented by AOX treatment 3 days after nerve damage (Figure 8I). *In situ* observation of oxidative stress using a ROS-sensitive dye shows a clear appearance of positive signal in sciatic nerve axons 24 hours after nerve damage (Figure 8J), consistent with the increase in lipid peroxidation (Figure 8I).

Taken together, our data demonstrate that in mice, as in *C. elegans*, axonal degeneration depends on the anti-oxidative cellular capacity, which can be enhanced directly by antioxidant treatment or indirectly by dietary restriction.

## Discussion

The Insulin/IGF-1-like signaling (IIS) pathway is implicated in a number of cellular processes, including aging and stress resistance; its known role in the regulation of antioxidative mechanisms has been associated with the protection of diverse tissues from chemical and genetic insults [18]. In this work, we show for the first time that downregulation of the IIS pathway *in vivo* prevents degeneration of both neuronal somas and axons in a cell autonomous fashion. We then show in mice that systemic treatments that increase the antioxidative cellular state protect axons from Wallerian degeneration, a process that has been associated with the progression of several neurodegenerative conditions.

Touch receptor neuron degenerating by the persistent activity of the MEC-4d degenerin channel is an excellent model to study mechanisms of neuronal degeneration. We improved the model by including detailed morphological description of different neuronal compartments, including somas and axons, and a functional readout of neuronal integrity. To date there is no *in vivo* electrophysiological data about MEC-4d functionality in the touch receptor neurons. We hypothesize that MEC-4d channels, despite exhibiting a persistent sodium current, can be further activated. A deeper exploration on MEC-4d properties could be performed by *in vivo* electrophysiological recording of TRNs in *mec-4d* mutants as in O'Hagan *et al.* [48] under conditions that prevent axonal degeneration.

Degeneration by *mec-4d* expression is mechanistically overlapping to mammalian neuronal degeneration, as its depends on extracellular Ca^2+^ ions [14], is delayed by CsA treatment [7], and is protected by overexpression of NMAT-2, the *C. elegans* homologue of mammalian NMNAT-2 (Figure 7D–7G).

Even though in the *C. elegans* model of neuronal demise presented here the relationship between soma and axonal degeneration is not distinguished, several evidences suggest that the pro-degenerative stimuli act directly in the axonal compartment, including the expression of MEC-4d in the axon (Figure 2I), their early degeneration compared to somas (Figure 1E–1F) and the axonal protection conferred by NMAT-2 overexpression (Figure 7F), a manipulation known to directly affect axon degeneration in other systems.

### Downregulation of the IIS pathway and dauer formation prevent neuronal degeneration

Lack of nutrients signals the downregulation of the IIS pathway in *C. elegans* and the formation of the dauer larvae, a diapause state that can last for up to four months [18], [49], [50]. Entry into dauer prevented neuronal degeneration as long as worms were kept in diapause. Exit from dauer resumes the degeneration program of TRNs. Protection from neuronal degeneration during dauer was not exclusive of the *mec-4d* triggered death: nematodes with the *deg-1* mutation, which kills the PVC cells, also were protected from neuronal demise during diapause. This speaks of a broad mechanism of neuronal protection in the dauer state, which represents a novel characteristic of this diapause condition.

Downregulation of the IIS pathway by *daf-2* mutation protected TRN from degeneration to a great extent, comparable to the dauer-dependent protection, suggesting that the mechanism responsible for neuronal protection in dauer is related to this pathway. By using neuronal-specific RNAi we demonstrated that protective mechanisms of the IIS pathway are activated in a neuronal autonomous fashion (Figure 4D–4E).

Downstream of DAF-2, the transcription factors DAF-16/FOXO and SKN-1/Nrf2 activate different mechanisms of oxidative stress surveillance. DAF-16 targets SODs and catalases while SKN-1 activates the detoxification phase II genes such as *gcs-1* [30]. Our results demonstrate that AVM protection from *mec-4d* induced degeneration is at least in part due to the transcriptional activation of DAF-16 downstream of DAF-2 in a neuronal autonomous fashion (Figure 5A–5B). Consistently, the protection granted by DAF-2 downregulation requires the expression of the antioxidant enzymes SODs and catalases (Figure 5C–5D). It is likely that reduction of cellular oxidative stress by activation of DAF-16/FOXO represents a neuroprotective mechanism in part by its ability to inhibit axonal degeneration, preventing the consequent demise of the neuronal soma. At the same time, the expression of organelle specific anti-ROS systems (*i.e.* SODs and catalases) as a result of DAF-16 activation may simultaneously prevent soma and axonal demise.

### Systemic antioxidant treatment and intermittent fasting delay neurodegeneration in worms and mice

There is a good correlation between factors that promote longevity and those that we have found here to prevent neuronal degeneration [51]. Likely these are processes that rely on mechanisms that preserve the cellular integrity through lowering, for example, oxidative stress.

Caloric restriction (CR) decreases oxidative stress in several tissues [41] and in mammals, reduces insulin levels with a concomitant nuclear translocation of FOXO and activation of its target genes [52]. Several studies have shown that caloric restriction increases life expectancy and is beneficial for healthy aging of the nervous system [52], [53]. CR also reduces brain damage in models of focal cerebral ischemia [54] and Parkinson's disease [55].

In our work, both adult worms and mice subject to CR showed delayed axonal degeneration. CR and reduced IIS are believed to exert their effects by a decrease in cellular oxidative damage [41]. CR causes a decrease in energy expenditure, ROS production and oxidative damage, while downregulation of the IIS increases cellular antioxidant defenses, which elevates resistance to oxidative stress [56]. CR has the advantage of being an intervention that is readily applicable to humans [57]–[59]. Importantly, these acute treatments can be implemented once organisms reach their fertile age with very short or even no pretreatment in the case of *C. elegans* and mice, and rendering impressive results both by genetic pro-degenerative triggers (worms, degenerin activation) as well as mechanical damage (mice, nerve damage).

Although the mechanisms by which CR increases resistance of cells to age-related pathology are not firmly established, two not mutually exclusive possibilities have been suggested: a reduction in mitochondrial oxyradical production by lowering metabolic flux [60] and activation of cellular antioxidant mechanisms [61]–[64] by an hormetic cellular response after a mild-stress associated to intermittent induction of mitochondrial metabolism and sub-lethal ROS production [65].

### Integrative model for ROS–dependent neuronal degeneration

Degeneration of TRN somas by *mec-4d* expression has been studied as a model of necrosis [66]. Calcium increase, activation of intracellular proteases (including calpains and cathepsins) and autophagy, are intermediary steps leading to vacuolation of TRN somas, which are later engulfed by neighboring cells in a *ced-2*, *5 and 10-*regulated process [67]. Our results demonstrate a critical role for ROS in this process, which can be controlled by manipulating the IIS pathway. This, together with its *nmat-2* dependence, better defines this necroptotic death mechanism of neuronal somas. We have further demonstrated that neuronal degeneration in worms and mice also depends on ROS production, which can be counteracted by genetic or pharmacological means, as well as reduction in the metabolic burden to the organism by CR.

**Figure 9:**
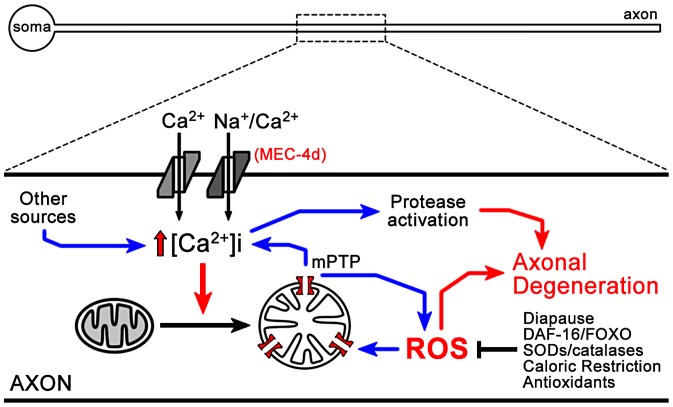
Model of ROS dependent neuronal degeneration. Opening of the MEC-4d channel results in an increase in intracellular calcium, which together with calcium contribution from other sources leads to mitochondrial dysfunction, ATP depletion and ROS generation, which enters into positive feedback loops, finally leading to activation of proteases in somas and axons. In dauer and *daf-2* mutants, anti-ROS systems are elevated, decreasing mitochondrial dysfunction and blocking neuronal degeneration in dauers.

Based on our results and published data, we propose a model in which opening of the MEC-4d channel results in an increase in intracellular calcium, which together with the endoplasmic reticulum calcium contribution [14], leads to mitochondrial dysfunction, ATP depletion and ROS generation, which enters into positive feedback loops, finally leading to activation of proteases in somas and axons. Importantly, the presence of axonal beading in *mec-4d* worms suggests that axonal transport defects might represent an early event triggering degeneration, in agreement with recent data [68]. In both dauer and *daf-2* mutants, anti-ROS systems are elevated, decreasing mitochondrial dysfunction and blocking neuronal degeneration (Figure 9).

Accumulative stress with age just as a consequence of living may resemble the acute effect caused by damage to neurons. Factors such as DAF-16/FOXO integrate and orchestrate a response to oxidative stress provoked by either processes being physiological or pathological. Since neurons are mostly irreplaceable, the nervous system is particularly susceptible to aging and injury and may profit from an intrinsic ability to detoxify itself that can be exploited for therapeutic purposes.

## Methods

### *C. elegans* growth and maintenance

*C. elegans* wild type (N2), mutants (*daf-2(e1370)III*, *deg-1(e38)X*, *mec-4d(e1611)X*) and transgenic strains [TU2773 {*uIs31*(*P_mec-17_mec-17::gfp*); *mec-4d(e1611)X}*, TU3755 {*uIs58(P_mec-4_mec-4::gfp)*}, VM484 {*akIs3(P_nmr-1_nmr-1::gfp)*}] were grown as previously described [69]. For time course experiments we synchronized worms by collecting the newly hatched L1 two hours after washing off a plate with mixed stage worms. Groups of 30 to 50 worms were gently collected in M9 using a mouth pipette and placed in new plates with food to be examined at the appropriate times. All experiments, unless noted were done at 20°C.

### Expression constructs and transformation

A 1 kb fragment 5′ from the start of translation of the F26H9.4 (*nmat-2*) gene was amplified from genomic DNA using the following primers AAAAGGATCCATGAAACGAGTCGCTCTTCTTGC and TTTTGGATCCATTTTCTGATACAGATTATTCTCCC, that introduced 5′ and 3′ BamHI sites. The resulting PCR fragment was cloned between the *mec-18* promoter and *yfp* coding sequence in TU#739 [70] to create WCH#19. We generated transgenic nematodes by microinjection [71] of 10 ng/µl of WCH#19, 30 ng/µl of pCW2.1 (a *ceh-22::gfp* plasmid [72]) and 60 ng/µl of pBSK (Stratagene) as filling DNA.

### Transgenic strains generated

WCH1 contains *wchEx1*, an extrachromosomal array containing *P_mec-18_nmat-2::yfp*; *P_ceh-22_gfp* expressed in wild-type worms. *wchEx1* was crossed into *mec-4d(e1611)X* to create WCH2. WCH4 contains *uIs31(P_mec-17_mec-17::gfp)* in a *daf-2(e1370ts)III*; *mec-4d(e1611)* double mutant. WCH6 contains *uIs71(P_mec-18_sid-1;P_myo-2_mcherry)* and *uIs31(P_mec-17_mec-17::gfp)* arrays in a *sid-1(pk3321)V*, *mec-4d(e1611)X* double mutant. *uIs58(P_mec-4_mec-4::gfp)* was crossed into *mec-4d(e1611)X* animals to create WCH32, and *akIs31(P_nmr-1_nmr-1::gfp)* was crossed into *deg-1(u38)X* mutants to create WCH33.

### Criteria for touch neuron integrity

To assess touch neuron morphology, worms were immobilized by placing them on a drop of 20 mM sodium azide directly on a 2% agarose pad. All worms were observed under a 100× objective, in an upright fluorescent microscope (Olympus) in a 20 minutes range to avoid damage for long exposure to sodium azide.

We used the *uIs31* strain (*P_mec-17_mec-17::gfp*) as a control for the time of appearance of the postembryonic cells (AVM and PVM). We established the following morphological categories for somas and axons: Somas, SoW: soma wild-type, SoV: soma vacuolated, swollen several times the wild type size; SoØ: soma absent. Axons, AxW: axon wild-type; AxB: beaded axon of wild type length; AxT: axon truncated, of smaller length than wild type and sometimes detached from soma; AxØ: axon absent. When the axon was truncated and beaded it was scored as AxT.

Since we used *gfp* expression as the only morphological measure of neuronal integrity, there is a possibility that neuronal degeneration assessed by this mean follows a different degeneration dynamic than when studied by other techniques. The fact that TRN axons degenerate in *mec-4d* mutants, has been solidly established by electron microscopy analysis [13].

### Touch sensitivity

Worms were touched gently with an eyebrow hair [19] with alternative anterior and posterior touches (five times each) to determine an average response. These experiments were done in triplicates for each time point. Because we scored the functionality of the AVM touch neuron, which participates in the anterior touch circuit, only anterior responses (5 in total) are reported here. When AVM is intact (as in *mec-4d* dauer) the maximum anterior response is of three touches. Only worms that moved when prodded with a platinum wire pick or responded to the nose touch, were assayed. To test the function of the PVC neurons, a posterior (tail) touch was performed in *deg-1(u38)* animals at various times. *deg-1(u38)* animals have normal locomotion and are sensitive to anterior touch, which permits unequivocal interpretation of a negative posterior touch response.

### Quantification neuronal degeneration in dauer larvae

Mixed-stage worms were grown until all food exhausted to induce the formation of large amounts of dauer [73]. Plates were observed periodically every 24 hours for the appearance of dauer larvae. The first day dauers appeared on the plate was considered the day zero of dauer and from then a week or a month was counted. At the appropriate time, all worms were collected in M9, centrifuged and incubated in 1% SDS for 30 minutes to eliminate all but dauer larvae [21]. Quantification of AVM integrity was carried out in the same way as non-dauer worms (see above).

*Touch tests*: wild type (N2), *uIs31;mec-4d(e1611)* or *deg-1(u38)* SDS treated dauers were placed on a plate without food and allowed to disperse before touching them. All strains were touched 10 times in a head to tail fashion. In *deg-1(e38)* mutants, which are characterized by tail insensitivity due to death of the PVC neuron, we scored the posterior response [32]. *mec-4d(e1611)* mutants were assessed for the rescue of the anterior response, as a measure of functionality of the AVM. Responsive *mec-4d(e1611)* worms were separated then from non-responsive and both groups observed separately in the microscope for AVM integrity.

*Dauer recovery*: One week and one month dauers were treated with SDS, plated on food and scored every 24 hours for 120 hours. Each time point was done in triplicates of 30 worms each.

### Temperature-sensitive experiments

Experiments to restrict the expression of *daf-2* in CB1370 [*daf-2(e1370ts)III*] and WCH4 (*daf-2(e1370ts)III; uIs31*(*P_mec-17_gfp*); *mec-4d(e1611)X* were done at 25°C. L3–L4 worms grown at 20°C were passed to 25°C and allowed to grow for 2 days and lay eggs. We synchronized worms by washing off the plates and leaving eggs that remained attached to the bacterial lawn or agar. Two hours later, 50 newly hatched larvae were picked with a mouth pipette to each new plate and kept at 25°C. Each time point was done in triplicates of 30 worms each.

### Cell-autonomous RNAi

Bacteria expressing dsRNA from the Ahringer library [74], [75] were grown on LB plates supplemented with ampicillin at 37°C overnight. Next morning a large amount of bacteria was inoculated in LB liquid supplemented with ampicillin and grown for 6–8 h. The resulting culture was seeded onto 1-day-old NGM-IPTG-carbenicillin plates and allowed to dry for 24 hours. For combinatorial RNAi, each bacterial culture was grown separately overnight in liquid LB. Before seeding the plates, the contents of each culture were spun down, concentrated in half the volume and mixed in equal parts to reconstitute the same concentration used in single RNAi experiments. We added 30–50 newly hatched L1 with a mouth pipette onto each plate and grew them at 20°C.

### Intermittent fasting

We designed an intermittent fasting protocol with 12 hour-shifts that consisted of 3 hours with *ad libitum* food followed by 9 hours without food, for 72 hours at 20°C. We began with 1 month-old dauers, selected by SDS treatment as detailed above. After the 3 hours on food, worms were washed off using M9 medium and spun down for 3 minutes at 2000 rpm. Three washes were performed to eliminate bacteria. For the 9 hours without food, worms were placed on NGM plates carrying ampicillin to avoid any growth of *E. coli* OP50.

### *C. elegans* drug treatment

Drugs were administered in the food, by mixing the appropriate concentrations of the chemical with *E. coli* OP50. New plates were made every 24 hours to keep the chemical fresh, and worms changed by hand picking on the next morning. The scoring was always done at 72 hours post-hatching. The following drugs and concentrations were used; EGTA (Sigma, 50 mM), cyclosporin A (CsA, LC Laboratories, 50 µM), trolox (Sigma, 100 µM), ascorbic acid (Sigma, 50 mM).

### Mice handling, treatment, and surgery

Mice of the wild-type (WT, C57BL/6J) strain were obtained from the University animal house. Transgenic YFP (B6.CG-T6, THY1-YFP) mice strain were obtain from Jackson laboratories (USA). Adult mice 11-week old, weighting 20–25 g were used. Experiments with mice followed protocols approved by the Institutional Animal Care and Use Committees and complied with NIH guidelines.

Ascorbic acid was diluted in the mice drinking water at a concentration of 0,5 g/L. A pretreatment of 10 days was performed prior to the nerve crush. As mice consumes between 4–5 ml of water per day (as measured daily in our experimental groups), the systemic dose of ascorbic acid corresponds to 2,5 mg/g per day. We used the body surface area (BSA) normalization method [76] to calculate the mice dose administration of ascorbic acid from the human dose, in which the Human Equivalent Dose (HED, 10 mg/kg) is multiplied by the ratio between the Km factor of the human (37) and the Km factor of the mouse (3) (Office of New Drugs, 2005), resulting in an animal dose of 123,33 mg/kg. Considering a 20 mg mouse the daily dosage of ascorbic acid given was 2,47 mg/animal, dissolved in its drinking water according to an intake per day of 5 ml/animal (data not shown). No difference in the intake of water with or without ascorbic acid was observed. For dietary restriction, mice were fasted *ad libitum* every other day with regular mice food for 10 days before nerve crush. Mice weights were daily measured in all experimental groups with no significant differences between them, except in the dietary restricted group, which shows a regular decrease of 3 g after the non-feeding day, but with a net weight increase comparable to the other experimental groups.

For nerve crush, mice were anaesthetized with Avertin (0.37 mg/g) and the sciatic nerve was exposed at the mid-thigh and crushed three times for 5 seconds each, using Dumont #5 forceps, the crush site was marked with graphite powder applied in the forceps and the wound was closed using surgical clips.

### Immunofluorescence and electron microscopy of sciatic nerves

For immunofluorescence analysis, sciatic nerves were fixed by immersion in 4% paraformaldehyde in 0.1 M phosphate buffer saline (1× PBS, pH 7.4) for 1 hour. Followed by 3×10 minutes washes in 1× PBS, sucrose gradient (5%, 10%, 20% in 1× PBS) and then embedded in OCT (Sakura Finetek). Cryostat sections from the middle of the explants were cut transversely at 10 µm thickness and mounted on Superfrost Plus slides (Thermo Scientific). Sections were washed in 1× PBS for 10 minutes and then blocked/permeabilized in 0.1% Triton X-100, 2% fish skin gelatin (Sigma Aldrich) in 1× PBS for 1 hour at room temperature (RT). Sections were incubated in primary antibodies in blocking/permeabilizing solution overnight at 4°C, washed in 1× PBS 3×10 minutes, and incubated in secondary antibodies for 2 hours at RT. Sections were washed 3×10 minutes in 1× PBS and mounted in Vectashield (Vector laboratories). Number of axons per area of nerve tissue was assessed in images of neurofilament-immunostained explant sections using the particle analysis macro of ImageJ.

The following antibodies were used for immunofluorescence analysis: rabbit anti-neurofilament heavy chain (Sigma-Aldrich, #N4142) at 1∶1000; chicken anti-neurofilament medium chain (Chemicon International, #AB5753) at 1∶2000; donkey anti-rabbit TRITC (Jackson Immunoresearch Lab. Inc., #711-025-152) at 1∶300; and, goat anti-chicken FITC (Invitrogen, #A-21449) at 1∶300.

For EM analyses, nerves were fixed overnight by immersion in 2.5% glutaraldehyde, 0.01% picric acid, 0.1 M cacodylate buffer (pH 7.4). Nerves were rinsed in the same buffer, immersed in 1% OsO_4_ for 1 hour followed by in block incubation with 2% uranyl acetate for 2 hours. Nerves were dehydrated with a graded series of ethanol, propylene oxide and infiltrated with Epon (Ted Pella Inc.). Ultrathin sections from the middle of the explants were contrasted with 1% uranyl acetate and lead citrate. Grids were examined with a Philips Tecnai 12 electron microscope operated at 80 kV. Negative films were developed and scanned.

### *Ex vivo* axonal degeneration

Sciatic nerve explants were performed as previously described [7]. Briefly, sciatic nerve segment were dissected from adult mice and cultured in Neurobasal medium supplemented with 2% B27 (Invitrogen), 0.3% L-glutamine, and 1% streptomycin/penicillin. C57BL/6J nerves were incubated in culture medium alone (Vehicle) or with trolox (Sigma-Aldrich, 1 mM). The explant culture medium was daily replaced.

### Lipoperoxides content measurement

Sciatic nerves were frozen in liquid nitrogen and homogenized in 10% trichloroacetic acid (TCA). The homogenate was subjected to centrifugation at 10.000 rpm for 10 min at room temperature, the resulting supernatant was used as the lipoperoxide fraction. Oxidized lipids were detected by mixing the supernatant with a reaction buffer (0,5% thiobarbituric acid and 20% TCA). The reaction solution was then incubated at 100°C for 30 min and thereafter the absorbance was measured at 512 nm. Lipoperoxide content was determined using the extinction coefficient of 155 mM^−1^×cm^−1^ and normalized to the starting nerve dry weight (modified from reference [77]).

### ROS detection in *C. elegans* and mice

Worm embryos were collected after hypochlorite treatment and embedded in OCT (Sakura Finetek) without a fixation step. Cryostat sections were cut at 10 µm thickness and mounted on Superfrost Plus slides (Thermo Scientific). Sections were washed in 1× PBS for 10 minutes and then incubated at room temperature in the fluorogenic probe CellROX Deep Red Reagent (Invitrogen, C10422) at a final concentration of 20 µM for 30 min. After washing in 1× PBS, sections were fixed in 4% paraformaldehyde in 1× PBS for 10 minutes followed by 3×10 minutes washes in 1× PBS. Sections were mounted in DAPI-containing Vectashield (Vector laboratories).

Sciatic nerves from wild type and YFP expressing mice were dissected at different times post injury and the perineurium was removed under a dissecting microscope. Nerve fiber bundles were attached to 3-aminopropyltriethoxysilane (Sigma-Aldrich) coated slides and incubated with the fluorogenic probe CellROX Deep Red Reagent (Invitrogen, C10422) at final concentration of 20 µM for 1 h at 37°C. After washing in 1×PBS, nerve fibers were mounted in Fluoromount-G (Southern Biotech) and visualized by confocal microscopy.
